# Sarcoidosis: Psychotherapy and Long-Term Outcome—A Case Report

**DOI:** 10.1155/2012/232491

**Published:** 2012-04-03

**Authors:** Giancarlo Trombini, Elena Trombini

**Affiliations:** Department of Psychology, University of Bologna, Viale Berti Pichat, 5-40127 Bologna, Italy

## Abstract

Sarcoidosis is a systemic, inflammatory disease of unknown aetiology, influenced by stressful life events and associated with a high incidence of alexithymic personality traits, and of depressive symptoms. The medical literature on sarcoidosis has called for a psychotherapeutic intervention to modify the perceived state of disease, the influence of stressful events and the depressive condition. Few studies have described cases treated with psychotherapy, and no information is available on its long-term outcome. We present the case of a patient with chronic sarcoidosis and periodical reacutizations with constantly pathological ESR. Twenty-four years after the diagnosis, a dynamic supportive-expressive psychotherapy for psychosomatic alexithymic patients was added to the medical therapy. At the beginning and at the end of the psychotherapy, and for the long-term outcome evaluations, Kellner's symptom questionnaire (SQ) was used to investigate psychological distress. The SQ scores, initially pathological, were normal at the end of the psychotherapy and for the following three years. Psychotherapy, without antidepressive drugs, resolved the depression. The depressive symptoms disappeared, along with the normalization and stabilization of the ESR. After three years, the outcome was positive. This is the first study describing a successful psychotherapy and its long-term outcome on a patient with sarcoidosis.

## 1. Introduction

During the last 10 years, the medical literature has repeatedly called for a psychotherapeutic intervention for patients affected with sarcoidosis [1–4], aimed at modifying the perception of the state of disease, the emotional influence of stressful events, and the depressive state. However, in literature there is a lack of studies on clinical cases treated with psychotherapy, and no information is available on the long-term outcome of this kind of treatment.

Sarcoidosis is a systemic and inflammatory disease of unknown aetiology [5], which commonly affects the lungs, the skin, the lymph nodes, the liver, and the nervous system. This disorder can be chronic, progressive, and self-limiting.

Regarding the aetiopathogenesis, stressful life events like getting married, starting a job, and changes in responsibilities at work have been shown to significantly influence the onset and the worsening of the disease. Among patients with sarcoidosis, a high incidence of alexithymic personality traits has also been evidenced [6–9].

The more severe forms may have profound effects on the quality of life of the patients [7], and a high prevalence of depressive symptoms was found in affected patients [10]. It has been suggested that clinicians should consider a psychological intervention with or without antidepressants according to whether significant depressive symptoms are identified [1].

The present paper describes the case of a patient affected by chronic sarcoidosis with periodical reacutizations treated with a dynamic supportive-expressive psychotherapy.

This is the first study describing a psychotherapy and its long-term outcome on this kind of patients.

Dynamic psychotherapy has already been described elsewhere [11, 12] in its two aspects, supportive and expressive.

Regarding the supportive aspect, Freud himself, in An Outline of Psycho-Analysis (1938), insisted on the necessity of reassuring and encouraging patients to amplify their mental life. He was aware of the importance of the cognitive aspects as a support in overcoming anguish.

Luborsky and Luborsky [12] affirm that the support does not directly aim at producing insight, even though it can favor it. It is directed at building a setting and a relationship to give security to the course of the cure.

An important condition for this support is the structure of the treatment itself, expressed by regular meetings and by the feeling, shared by both the therapist and the patient, of cooperating in meeting the patient's objectives. Lastly, fundamental to providing support to the patient are the therapist's listening skill and his/her empathic behavior, which must be encouraging but not judging, while examining and facing the patients' daily problems.

Regarding the expressive aspect, it is the central element differentiating dynamic psychotherapies from other forms of treatment. The expressive activity transforms the psychotherapy in a process of discovery of the self. It encourages the patient to express feelings and thoughts, allowing the patient-therapist couple to reflect and to explore meanings, identifying the possible sources of the psychic suffering. Lastly it underlines the relational modalities that the patient must learn to be in charge of.

## 2. Case Report

Mr G, a 48-year-old Italian man, manager of a small firm, married and father of two children, was diagnosed with sarcoidosis 24 years ago. The diagnosis, based on radiological and histological findings, was made at Ospedale Maggiore in Bologna. Tuberculosis, cryptococcosis, histoplasmosis, and other infective diseases were excluded by the laboratory analysis. The disease concerned his skin and his lungs, with the characteristic noncaseous granulomas. The main symptoms were an erythema nodosum to the legs and dyspnoea. The disease did not affect the central nervous system or other organs.

The patient was treated with corticosteroids, with different dosages according to the clinical condition. The physician noticed a state of depression and thus requested an evaluation from a clinical psychologist.

During the clinical interview the patient showed an alexithymic communication style, with limited speech, a pessimistic approach to the future, and social withdrawal. He worried about appearing idle at work, and spending little time with his family, with whom he felt isolated and not respected. He criticized himself and felt mortified when going against his own limits in his professional activity. He felt helpless that he had failed to meet his own expectations and that he was unable to cope with his pressing problems. This evidenced a state of demoralization, particularly frequent in medically ill patients [13].

Moreover, he suffered from dyspnea, and, every 3-4 months, the sarcoidosis nodule on his right leg worsened, as it had been doing for the last ten years. The sarcoidosis manifested itself six months after the patient started working and got married. These events coincided with the onset of the oncological disease of his father, who died three years later. At the time of his father's death, the patient had just started an entrepreneurial activity. The patient was therefore left without the important psychological support of his father. During this period the wife's depressive disorder, which had already appeared five years before, returned, with the onset of the oncological pathology of her mother, who died the year after the death of the patient's father.

A minor depressive disorder was diagnosed, according to DSM-IV-TR [14], based on the following aspects: depressed mood, feelings of low self-esteem, and difficulties in making decisions. From the anamnesis these research criteria were met during the last decade.

To the medical therapy, consisting of 5 mg/die of Prednisone, a supportive-expressive weekly psychotherapy session was associated, according to the technique introduced by Trombini for psychosomatic patients with alexithymic traits [15, 16]. At the beginning and at the end of the psychotherapy, and for the long-term outcome evaluations, the patient's psychological distress (anxiety, depression, somatization, and hostility) was investigated using the Italian version of the Symptom Questionnaire (SQ) by Kellner [17]. The SQ is a widely used instrument in psychosomatic assessments and in the test-retest control of psychotherapies [18].

The psychotherapy lasted three years.

## 3. Results

It is important to underline that during the course of the illness the patient had always shown pathological ESR values (always ≥26), at medical check-ups. At the beginning of the psychotherapy the value of the ESR was pathological (ESR value: 26). After 12 months the ESR was normal (ESR value: 9), and after 26 months it was stabilized (ESR value: 7).

At the beginning of the psychotherapy, the FEV1 was 81,8% and the FVC was 88,9%, and after the psychotherapy, the FEV1 was 95,4% and the FVC was 99,4%.

At the beginning of the psychotherapy, the usual worsening of the nodule in the right leg was expected to happen, as it had already appeared three months before, but it did not present itself.

At the beginning of the psychotherapy the patient showed the following SQ scores : anxiety = 8 (moderate distress), depression = 14 (severe distress), somatization = 9 (moderate distress), and hostility = 3 (in the range of normality).

After three years, the psychotherapy was brought to an end, and the patient showed the following SQ scores, all in the range of normality : anxiety = 5; depression = 4; somatization = 3; hostility = 3.

The patient's medical and psychological conditions were evaluated during the three years that followed the psychotherapy. The clinical condition was stable, and the patient maintained a state of well-being, as evidenced by SQ scores in the range of normality after one (anxiety = 3, depression = 4, somatization = 3, and hostility = 0), two (anxiety = 3, depression = 4, somatization = 1, and hostility = 0), and three (anxiety = 3, depression = 4, somatization = 2, hostility = 0) years.

During the psychotherapy, the patient's social withdrawal disappeared, as he managed to get in touch with new clients. He showed more self-esteem and participated more actively in his family's life, from which he no longer felt excluded. At the end of the psychotherapy, having resolved his depressive disorder, he said: “If I had not had these sessions I would never have got over my depression, which would have been really frightening.”

## 4. Discussion

The paper presented a case of sarcoidosis in which the psychodiagnostic evaluation of the patient evidenced, in line with literature [1–4], stressful life events preceding and contemporary to the onset of the disease, alexithymic personality traits, and depressive symptoms. Specifically, the following stressful life events were identified: the beginning of a job, a marriage, a severe illness and consequent death of the father, a change of work responsibilities, and his wife's episode of acute depression.

Literature has underlined the importance, for clinicians, of diagnosing and treating depression in patients affected by sarcoidosis with antidepressants and/or psychological interventions [1, 10, 19].

Moreover, the literature regarding medically ill patients has underlined that the presence of conditions of demoralization represents a common reason for subjects seeking psychotherapeutic treatments [13].

Therefore, a psychological intervention was proposed to the patient. It consisted in a dynamic supportive-expressive psychotherapy used for psychosomatic alexithymic patients [15, 16]. The supportive aspect helped the patient progress within the treatment, allowing him to overcome the limitations which had conditioned his life. The expressive aspect allowed the patient to give a psychological meaning to his behavior.

The psychotherapy resolved the depression without the use of antidepressive drugs. In fact the patient overcame his social withdrawal and showed a more confident attitude towards the future. He started speaking about his family and not just about work, finding more time to dedicate to the former and sharing their relational life. It is also relevant that, after a long period of illness, the introduction of the psychotherapy did not only resolve the patient's depression, but the disappearance of the depressive symptoms also coincided with a clinical improvement in the sarcoidosis. This was evidenced at a symptomatological level, but also in the laboratory tests (normalization and stabilization of the ESR improvement in the dyspnea) and in the psychometric ones (normalization and stabilization of the SQ scores).

The outcome after three years was positive.

The dynamic supportive-expressive psychotherapy proves to be efficient both physically and psychologically with this kind of patients.

The combined medical-psychological approach, used in the present case, can be tested and extended to other patients affected with sarcoidosis. Further investigations could evidence the selection criteria for patients who might respond to psychotherapy and the possible need of associated psychopharmaceuticals.

Lastly, the incidence of further severe life events must not be forgotten, as they might modify the attained state of well-being and might favor a reappearance of sarcoidosis.
